# Exposure to Sodium Fluoride Produces Signs of Apoptosis in Rat Leukocytes

**DOI:** 10.3390/ijms11093610

**Published:** 2010-09-27

**Authors:** José Gutiérrez-Salinas, José A. Morales-González, Eduardo Madrigal-Santillán, Jaime Esquivel-Soto, César Esquivel-Chirino, Manuel García-Luna y González-Rubio, Sigrit Suástegui-Domínguez, Carmen Valadez-Vega

**Affiliations:** 1 Laboratorio de Bioquímica y Medicina Experimental, División de Investigación Biomédica, Centro Médico Nacional “20 de Noviembre”, ISSSTE, México, D.F., México; 2 Área Académica de Medicina Instituto de Ciencias de la Salud, Universidad Autónoma del Estado de Hidalgo, Ex-Hacienda de la Concepción, Tilcuautla, 42080 Pachuca de Soto, Hgo., México; E-Mails: jmorales101@yahoo.com.mx (J.A.M-G); eomsmx@yahoo.com.mx (E.M.-S.); m.valadezvega@lycos.com (C.V.-V.); 3 Facultad de Odontologia, Universidad Nacional Autónoma de México (UNAM), México, D.F., México; E-Mails: jaime_esquivel2003@hotmail.com (J.E.-S.); cesquivelch@gmail.com (C.E.-C.)

**Keywords:** sodium fluoride, apoptosis, leukocytes, p53, bcl-2, caspase-3

## Abstract

Fluoride is naturally present in the earth’s crust and can be found in rocks, coal, and clay; thus, it can be found in small quantities in water, air, plants, and animals. Therefore, humans are exposed to fluoride through food, drinking water, and in the air they breathe. Flouride is essential to maintain bone strength and to protect against dental decay, but if it is absorbed too frequently, it can cause tooth decay, osteoporosis, and damage to kidneys, bones, nerves, and muscles. Therefore, the present work was aimed at determining the effect of intake of sodium fluoride (NaF) as an apoptosis inducer in leukocytes of rats treated for eight weeks with 1 or 50 parts per million (ppm) NaF. Expression of p53, bcl-2, and caspade-3 were used as apoptotic and general metabolism indicators of leukocyte-like indicators of the (INT) oxidation system. Male rats were exposed to NaF (1 and 500 ppm) for eight weeks, and then sacrificed weekly to obtain blood samples. Expression of p53, bcl-2, and caspase-3 were determined in leukocytes by Western blot, and general metabolism of leukocytes was analyzed with a commercial kit. We found changes in the expression of the proteins described, especially when the animals received 50 ppm of NaF. These results indicate that NaF intoxication can be an apoptosis inducer in rat leukocytes treated with the compound for eight weeks.

## 1. Introduction

Fluoride is a chemical element that is very abundant on the earth’s surface and that is recognized as an element that can produce acute and/or chronic changes in humans when they are exposed to it. Fluorosis (intoxication by fluoride) is caused by acute or chronic ingestion of fluorides and is clinically characterized in humans by dental alterations, as well as alterations in the nervous and musculoskeletal systems [1].

At the cellular level, experiments conducted *in vitro* have demonstrated that application of fluoride can produce diverse metabolic changes, such as glycolysis inhibition, changes in membrane receptors, alteration in total energy balance, DNA breaks, and induction of apoptosis [2–5].

In this latter case, it has been postulated that apoptosis induced by an excess of fluoride is produced in part by the phenomenon associated with the production of Oxygen-derived free radicals (OFR) that, being highly reactive molecules, can induce changes in biomolecules such as proteins, lipids, carbohydrates, and nucleic acids [6].

It has been described that application of sodium fluoride (NaF) as the main fluoride source to cells under culture can induce an excess of p53, a protein expressed when there is damage to the DNA [7]. On the other hand, induction of apoptosis has been described in the epithelial cells of human lung cultured with growing amounts of NaF, as well as general changes in alveolar macrophages under culture [8,9]. Additionally, it has been described that incorporation of fluoride in the diet of animals under experimentation can cause liver damage expressed as induction in the expression of caspases (that are the last effectors of apoptosis), as well as of the protein bcl-2 (the protein activated during apoptosis) [10,11].

Conversely, it has been reported that in geographic zones where fluorosis is endemic, there is an increase in the presence of bacterial, viral, and parasitic infections in humans or in wild or farm animals that have been intoxicated with this type of compound when it is present in drinking water in concentrations exceeding the maximal dose recommended by the World Health Organization (WHO), which is <1 parts per million (ppm) of NaF [1,2]. In Mexico, acute and chronic fluorosis is present mainly in central-northern states in which the water presents NaF concentrations that exceed WHO health norms [1]. The presence of NaF in aquifers is very important because this compound is not filtered by the conventional water purification systems that are employed precisely to prepare water for human consumption [1–6]. Also, NaF dissolved in water has neither taste nor smell; thus, people can ingest great quantities of this toxin without being aware of it [1,6]. Once consumed, NaF can cause alterations in systems such as digestive, nervous, reproductive, and immunological [1,12].

As mentioned previously, individuals who present fluorosis are more susceptible to presenting bacterial, viral, and parasitic infections, which indicates that there can be a change in their immune system, in which leukocytes play a very important role as defense systems against these infections [1]. From another viewpoint, it is known that leukocytes in peripheral blood circulation that have completed their useful life present a natural process of apoptosis and are eliminated from the organism [1,4,8]. Experiments *in vitro* have demonstrated that leukocytes incubated in the presence of NaF present an increase in the expression of proteins p53 and bcl-2, which are considered markers of the presence of apoptosis [1,4–8] and which would indicate that this toxin can promote this type of phenomena in systems *in vivo*.

The objective of the present work was to describe the effect of NaF on the expression of proteins bcl-2, caspase-3, and p53 as indicators of apoptosis in rat leukocytes.

## 2. Materials and Methods

### 2.1. Chemicals and Animals

Male Wistar-strain rats (weighing an average of 250 ± 5 g) were obtained from Laboratorios Harlam-México and placed in individual containers under 12-h light-dark cycles with free access to food and water. Male rats were selected because it has been demonstrated that NaF exerts a more constant effect, because females present hormone cycles that affect the results [13]. All procedures were carried out according to the guidelines contained in the Regulation on Treatment of Animals for Surgery and Research of our institution, which is in agreement with the Federal Regulation Law for Research and Experimentation Animals (SAGAR, Mexico, 1999). Chemical reagents used were of analytical grade and of the best possible quality. They were obtained from Sigma Chemical Co. (St. Louis, MO, USA), Merck de México S.A., or from Mallinckrot de México, S.A.

### 2.2. The Treatment Protocol

The animals were treated according to established protocols in which experimental animals are exposed to known concentrations of NaF, which is added to their drinking water [12,14], and changes were monitored each week for the time assigned by the protocol.

The rats were divided randomly into the following study groups: (25 animals per group):

- Control group: Rats without any treatment that ingested food (Rat Pellet Chow; Purina, Inc., MO, USA) freely with water from containers of the liquid for human consumption (Electropura-brand drinking bottled water; The Pepsi Bottling Group México, S. de R.L. de C.V.). According to chemical analyses, this water presented a fluoride concentration of <0.5 ppm; thus, it is considered to fall within normal limits and has been reported as not interfering with the experiment.
- Experimental group 1: Rats to whose drinking water we had added a concentration of NaF equivalent to 1 ppm of fluoride and with free access to food [14].
- Experimental group 50: Rats to whose drinking water we added a concentration of NaF equivalent to 50 ppm of fluoride and with free access to food [14].

Once the treatment had been initiated, the rats of all groups under study were sacrificed every 2 weeks (five per group) for up to 8 weeks. During the entire treatment, water was substituted every 24 h with fresh water, and bromatological data were registered once per week.

### 2.3. Obtaining and Processing the Leukocytes

After anesthesia (ether vapor), the rats were sacrificed by decapitation as previously described [15]. Blood was collected in a recipient with an anticoagulant (EDTA) and placed in an assay tube as previously described [15–17]. Three volumes of histopaque (1077-1; Sigma Chemical Co.) and three volumes of whole blood were placed in an adequate tube, and this was centrifuged for 15 min at 1,500 rpm in a clinical centrifuge (Hermle Z-380, Germany). At the end of centrifugation time, the leukocytes that remained in intermediate phase were removed with a Pasteur-type pipette and were washed three-times with five volumes of cold phosphate isotonic saline solution (PBS, pH 7), then centrifuged at 1,500 rpm for 10 min., Once they were washed, the leukocytes were counted in a hemocytometer and the leukocyte concentration was adjusted to 2 × 10^6^ cells/mL.

### 2.4. Determination of the Metabolic Activity of Leukocytes

Quantitative evaluation of the metabolic activity of the leukocytes as an index of variability was performed with a commercial reagent kit (Leucognost®; Hematologie, Merck, Darmstadt, Germany) following the instruction guide provided by the manufacturer, which is based on the procedure reported by Lokaj *et al*. [18]. Briefly, the procedure was as follows: a sample of leukocytes was placed together with three volumes of Dulbecco solution with glucose (5 mM) and divided into two parts. One of these was incubated in the presence of 1% potato starch, 1% of dimethyl sulfoxide, and 0.1% of cloruro of 3-4-indophenyl-2,4-nitrophenyl-5-phenyl tetrazolium (INT; Sigma Chemicals). The remaining leukocytes were placed under the same conditions as those of the latter, but without starch. Both samples were incubated for 45 min at 37 °C under constant shaking. At the end of incubation, we added two-volumes of absolute methanol and shook this strongly for 3 min. The result was centrifuged at 5,000 rpm for 10 min and the supernatant was recovered; was and the absorbance read at 485 nm in a spectrophotometer (Jenway 6300; Cielo Vista, CA, USA). The metabolic activity of the leukocytes was expressed in nmoles of formasan/10^6^ cells according to the procedure described in the kit’s instruction manual.

### 2.5. Total Leukocyte Extract

A sample of leukocytes was diluted with equivalent parts (v/v) of cold lysis buffer (Tris, 0.01 M; sucrose, 0.255 M; EGTA, 0.3 mM; pH 7.4 containing protease inhibitors) and was homogenized with a sonicator (Ultra-Turrax 205; Thompson, Inc., USA) with three 2-min hit strikes, each. The homogenate was centrifuged at 10,000 rpm for 20 min. The supernatant was recovered and considered as the total leukocyte extract in which we determined the concentration of the total protein by the method of Lowry [19] utilizing bovine albumin as standard.

### 2.6. Expression of Proteins p-53, bcl-2, and Caspase-3

Analysis of the presence of apoptosis-indicator proteins in the leukocytes was conducted using the techniques of Western blot in acrylamide gels employing the general technique reported by Laemmli [20], followed by a nitrocellulose paper blot according to established methods [21].

Briefly, we carried out the following: samples of the total leukocyte extract were mixed with an equal volume of treatment buffer (2-mercaptoethanol 2 mM; SDS 10%; glycerol 1%; PBS, pH 8) and heated in a water bath for 3 min. At the end of incubation, samples of the reaction (100 μg of total protein) were placed in 12% acrylamide/0.1% bis-acrylamide-gel wells and submitted to a 150-mV current for 80 min in a vertical apparatus for electrophoresis (Bio-Rad, Ltd., Hercules, CA, USA) with a running buffer (Tris-base/SDS/glycine). At the end of the run, the gels were placed in an apparatus for electrotransfer (Bio-Rad, Ltd.) employing a nitrocellulose membrane as receptor, and a cold transfer buffer (Tris-base/glycine/methanol/SDS). Protein transfer was performed for 80 min at 100 mA, at the end of which the nitrocellulose membrane was dyed with a Ponceau red solution to determine localization of the proteins. Afterward, the membrane was washed three-times with PBS and incubated for 60 min at room temperature under continuous shaking in a blocking solution (5% powdered skim milk and 0.5% of Tween-20). At the end of incubation, the membrane was washed three times with PBS and was exposed to primary antibody (Santa Cruz, CA, USA) digested against the specific protein (p-53, bcl-2, or caspase-3), incubating this under continuous shaking for 70 min and with blocking solution. After this incubation, it was washed three times with PBS and exposed for 60 min to the peroxidase-labeled secondary antibody. The membrane was washed three times with PBS and we exposed the presence of the proteins with a solution of hydrogen peroxide (0.5%) and tetramethylbenzidine according to the instructions accompanying the commercial kit (Promega, USA) that is used for this purpose. Once the protein was localized, the membrane was scanned with Alpha Imagen ^TM^ equipment (V-3.3 1200; USA) with documentation and analysis system to determine, by means of absorbance (read at 640 nm), the apparent quantity of each protein per sample [22].

### 2.7. Statistical Analysis

Data analysis was conducted with the GraphPad Prism ver. 4.00 for Windows (GraphPad Software; San Diego, CA, USA) statistical software program with an Excel platform (Microsoft Co.). The results are expressed as averages ±Standard error (SE) for all study groups. The difference between groups was analyzed by two-tailed Analysis of variance (ANOVA), with Tukey post-test when applicable, taking a minimum value of *p* < 0.05 as statistically significant.

## 3. Results

One of the parameters taken into account for evaluating the general effect of a toxin on the integrity of a subject-under-study is performance of the determination of body weight during the treatment duration [12]. Table 1 depicts the body weight gain in the control group as well as in animals receiving 1 or 50 ppm of NaF at 8 treatment weeks. As can be observed, none of the NaF doses administered demonstrated a significant effect on body weight after 8 weeks of treatment. We also observed no significant differences in the amount of water consumed daily by the animals, and solely observed an increase in the weekly amount of NaF ingested by the group that received 50 ppm, which is entirely logical because this group consumed a greater concentration of the compounds in their running drinking water. During sacrifice of the animals, these were examined by a pathologist to denote whether there was some change in the morphology of the animals’ organs (especially liver and kidneys), and the result was negative.

**Figure 1:**
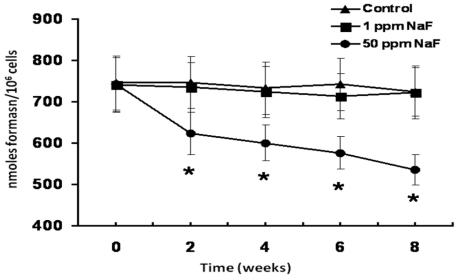
Time course of the metabolic activity of leukocytes obtained from the different study groups. Each point represents the average ± Standard error (SE) of five rats per time. Asterisks denote a statistical difference (*p* < 0.05) with regard to the control group.

Figure 1 shows a time course of the changes in the metabolic activity of the rat leukocytes treated with 1 or 50 ppm of NaF. As can be noted, in comparison with the control group, rats that received the 1-ppm dose of NaF did not produce significant changes in their metabolic activity. In contrast, the 50-ppm dose produced a significant decrease (*p* < 0.05) in this parameter from 2 weeks of treatment and the metabolic activity diminished by *ca.* 20% *(p* < 0.05) after 8 weeks of treatment (Figure 1).

**Figure 2:**
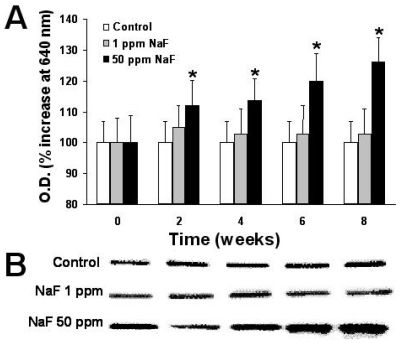
Time course of the expression of the p53 protein on the leukocytes of rats treated with 1 or 50 parts per million (ppm) of sodium fluoride (NaF) for up to up 8 weeks. (**A**). From Western blots scanned at 640 nm, the relative amount of protein is expressed in absorbance units throughout the treatment time. Each point represents those scanned in duplicate of five rats expressed as percentage of control ± Standard error (SE). Asterisks denote *p* < 0.05 with respect to the control group. (**B**). a representative image of a Western blot of the protein p53.

**Figure 3:**
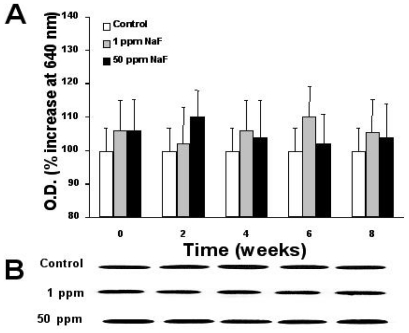
Time course of the expression of the bcl-2 protein on the leukocytes of rats treated with 1 or 50 parts per million (ppm) of sodium fluoride (NaF) for up to 8 weeks. (**A**). Western blots were scanned at 640 nm, and the relative amount of protein is expressed in absorbance units throughout the treatment time. Each point represents those scanned in duplicate of five rats expressed as percentage of control ± Standard error (SE). (**B**). a representative image of aWestern blot of the protein bcl-2.

**Figure 4:**
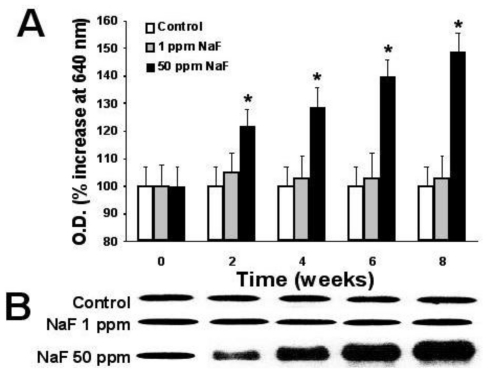
Time course of the expression of the protein caspase-3 on the leukocytes of rats treated with 1 or 50 parts per million (ppm) of sodium fluoride (NaF) for up to up 8 weeks. (**A**). Western blots that were scanned at 640 nm, and the relative amount of protein is expressed in absorbance units throughout the treatment time. Each point represents those scanned in duplicate of five rats expressed as percentage of control ± Standard error (SE). Asterisks denote a *p* < 0.05 with respect to the control group. (**B**). a representative image of a Western blot of the protein caspase-3.

Figures 2–4 depict the time course of the expression of proteins p53, bcl-2, and caspase-3 in the leukocytes of rats treated with the two doses of NaF and the determination, by scanning of the Western blots, of the relative quantity of protein, as described in the Materials and Methods section.

As can be noted, the group of rats that received the 50-ppm NaF treatment presented a statistically significant increase (*p* < 0.05) compared with the control group with regard to expression of proteins p53 (Figure 2) and caspase-3 (Figure 4) from two weeks of treatment. This increase was maintained throughout the eight weeks of the NaF treatment, and it is noteworthy that the 1-ppm dose of NaF presented no changes with respect to the controls in the expression of these proteins.

For its part, the bcl-2 protein exhibits a sustained pattern of expression throughout the treatment time in the control group, but it is not this way with the NaF-treated groups, whether with 1- or 50-ppm doses (Figure 3). In both groups, we observed an irregular pattern of expression each time that we carried out determination of this protein; however, differences in bcl-2 expression for both NaF-treated groups did not present a statistically significant difference among these or as compared with the control group.

## 4. Discussion

Ingestion of NaF incorporated into the drinking water of male rats with 50 ppm of NaF up to 8 weeks shows that their leukocytes present an important diminution in the expression of proteins p53 and caspase-3, as shown in Figures 2 and 4. These changes, mainly in the expression of the proteins p53 and caspase-3, can be indicative of an increase in apoptosis in this cell type that is induced by the presence of NaF, principally in the 50-ppm dose.

The data shown in Table 1, as well as the clinical data observed in the three study groups, demonstrate that treatment with both doses of NaF (1 and 50 ppm) does not produce the general visible alterations that have been reported as potentially toxic by other researchers [1,2,12]. On general inspection of the mucosa and teeth of all study groups, we observed no visible signs of fluorosis or any type of sign denoting intoxication according to previously established parameters [23,24]. The latter finding is in contrast with those of other investigators, who report the initiation of yellow speckling or mottle on teeth with NaF treatments ranging from 5–300 ppm and with a 6–21-week treatment time [1,2,12,25]. It is possible that the treatment time that we employed (8 weeks) is not sufficient for producing the visible alterations corresponding to fluorosis, in leukocytes as well as at the general level; however, the changes found in metabolic activity, as well as in the expression of proteins p53 and caspase-3, demonstrate to us that there are metabolic changes in peripheral blood leukocytes that are produced by our treatment. While, perhaps, these changes do not translate into visible clinical alterations, they do indeed denote a metabolic effect on leukocytes produced by NaF, above all at a 50-ppm dose.

Determination of the general metabolic activity of leukocytes has been considered an indicator of the normal functioning state of this type of cell [26]. The effect of NaF on the general metabolism of leukocytes has been reported previously [29]; however, these observations have derived from experiments conducted *in vitro* in which the cells have been previously isolated and cultured, which renders their interpolation to the entire organism difficult. From another perspective, in experimental models with laboratory animals, it has been reported that there is a decrease in the metabolic activity of their leukocytes when treatment with NaF lasts at least 10 weeks and up to 300 ppm of NaF in order to observe some type of change in this parameter [2,9,10,23–25].

This minimal time of observation has been based on the fact of recognizing that NaF-exposed animals should reach a degree of clinical intoxication for us to be able to observe metabolic changes that can be registered; however, in our treatment model, the animals present an important diminution in the metabolic activity of their leukocytes at two weeks of treatment with 50-ppm doses of NaF (Figure 1). This result could explain that, as mentioned previously, researchers procure that animals submitted to treatment with NaF present clinical or pathological signs of intoxication with this compound in order to consider that this element exerts effects on the organism. In this regard, we affirm that while animals do not present clinical or pathological signs of intoxication with NaF, they do present an alteration in their general metabolic activity, which can indicate metabolic sensitivity of leukocytes to this compound without the clinical expression of intoxication by the compound.

From another point of view, increase in the expression of proteins p53 and caspase-3 in the group of rats that received a 50-ppm dose of NaF indicates an increase of apoptosis in the leukocytes, because these proteins are considered indicative of an apoptotic process (caspase-3) due to cellular damage (protein p53) [11,27–29].

Protein p53 has been described as an indicator of DNA damage that is expressed after exposure to radiation or to a toxin [11,27–29]. While it is true that tissue that carries out a continuous replacement of cells due to a normal phenomenon of mitosis (as in the case of epithelial and mucosal tissues) presents a minimal expression of protein p53, it is also certain that this protein is expressed in excess when there is a toxin in the organism that alters genetic expression or damages the DNA [28,29]. Conversely, caspase-3 is a protease that degrades the cell’s cytoskeleton during a phenomenon that is exclusively apoptotic [30,31]. In this manner, leukocytes are cells that present a continuous replacement re-exchange in the circulation and that die naturally due to an apoptotic phenomenon [28–31]. Among effectors of apoptosis, we find caspases, whose most important representative is caspase-3, in that the latter is one of the main proteases that intervene in programmed cell death [29–31]. It has been described that exposure to a toxin can increase caspase expression as a reflection of an increase in the apoptosis of this tissue [11,29–31]. Due to what has been previously mentioned, we suppose that an increase in p53 and caspase-3 expression in rats exposed to a 50-ppm dose of NaF for eight weeks considerably augments the phenomenon of apoptosis in their circulating leukocytes. This affirmation is reinforced by the fact that the presence of NaF produces an asynchronic pattern of protein bcl-2 expression, as depicted in Figure 3. Protein bcl-2 has been considered an early indicator of the cell’s entrance into G1 phase of the cell cycle, and its expression presents synchronically throughout this phase [29–33]. It has been described that this protein can regulate the phenomenon of apoptosis when it is deregulated by a toxin or by exposure of the cells to radiation [31]. As illustrated in Figure 3, the presence of NaF at any of the tested doses produces asynchrony in bcl-2 expression, which, together with the data obtained for p53 and caspase-3, we can suggest that NaF induces an increase in the apoptosis of leukocytes in rat. It is noteworthy that the expression of the protein p53 is not in agreement with that of bcl-2 throughout the time of intoxication with NaF, which agrees with reports by other researchers, who found the same disparity among the expressions of apoptosis-related proteins [31,35]. The reason for this disparity in the expression of these proteins in our model of exposure to NaF is not known; therefore, additional studies are required to clarify this phenomenon, probably utilizing *in vitro* studies.

This increase in apoptosis due to the effect of exposure of the tissues or cells to NaF has been reported previously [8–10]; however, to our knowledge, there are no prior reports that describe the increase in apoptosis in circulating leukocytes in models *in vivo*.

The effect of NaF on circulating blood leukocytes that we have observed in this study gives us cause to suppose that this chemical compound can act as a toxic agent for these cells to the degree of inducing the process of apoptosis in them. This is of fundamental importance, because, as has been reported previously, in geographic zones where there is a high content of this toxin in the water, there is an increase of infectious diseases in animals as well as in humans, which makes us think that this compound causes important damage to the immune system [33,34].

Conversely, it is known that NaF is a compound that is widely utilized as an anticaries agent added to popular pastes and gels for dental hygiene in concentrations that can be as high as 1,500 ppm [14,34]. Although under normal conditions of use, toothpastes that contain NaF have only a brief time of contact with the organism (the time it takes to brush the teeth), it has been reported that children who are learning to brush their teeth and use fluorinated toothpastes tend to swallow this type of compound, which may provoke acute or chronic fluorosis, in addition to other metabolic problems [14,34].

## 5. Conclusion

According to the results obtained, we suggest that exposure of rats to NaF modifies the expression of p53, bcl-2, and caspase-3 and causes general metabolic changes toleukocytes, which are indicators of changes to normal pattern of apoptosis.

It is clear that additional investigations are required to evaluate more precisely the effects that NaF can exert in this type of cell; however, we are able to state that our study model can be useful for investigating in a more detailed fashion the effect of this toxin on leukocytes and on other cell and tissue types.

## Figures and Tables

**Table 1:** Body weight, water consumption, and sodium fluoride (NaF) doses in rats treated for up to 8 weeks with doses of 1 or 50 parts per million (ppm) of NaF. Results are expressed as averages ± Standard error (SE).

Treatment (*n*)	AIW (g)	AFW (g)	WCR (mL/day)	D-NaF (mg/kg/week)
Control (25)	252.13 ± 3.14	314.13 ± 5.39	29.05 ± 0.30	0
NaF 1ppm (25)	252.70 ± 3.32	312.23 ± 3.32	31.05 ± 0.32	0.868 ± 0.01
NaF 50 ppm (25)	251.65 ± 4.81	312.12 ± 5.21	30.48 ± 0.27	42.67 ± 0.38

*n*: number of rats per group; AIW: Average initial weight; AFW: Average final weight; WCR: Water consumption per rat; D-NaF: Dose of NaF.
